# Interplay between Polo kinase, LKB1-activated NUAK1 kinase, PP1β^MYPT1^ phosphatase complex and the SCF^βTrCP^ E3 ubiquitin ligase

**DOI:** 10.1042/BJ20140408

**Published:** 2014-06-26

**Authors:** Sourav Banerjee, Anna Zagórska, Maria Deak, David G. Campbell, Alan R. Prescott, Dario R. Alessi

**Affiliations:** *MRC Protein Phosphorylation and Ubiquitylation Unit, College of Life Sciences, University of Dundee, Dow Street, Dundee DD1 5EH, U.K.; †Molecular Neurobiology Laboratory, Salk Institute for Biological Studies, La Jolla, CA 92037, U.S.A.; ‡Division of Cell Signalling and Immunology, College of Life Sciences, University of Dundee, Dow Street, Dundee DD1 5EH, U.K.

**Keywords:** AMP-activated protein kinase (AMPK), AMPK-related kinase 5 (ARK5), cell cycle, degron, mitosis, Polo kinase (PLK) ubiquitylation, AMPK, AMP-activated protein kinase, CDK, cyclin-dependent kinase, CK1, casein kinase 1, Cul1, cullin 1, DMEM, Dulbecco’s modified Eagle’s medium, DTB, double thymidine block, Emi1, early mitotic inhibitor 1, GST, glutathione transferase, HA, haemagglutinin, HEK, human embryonic kidney, HRP, horseradish peroxidase, IKK, inhibitor of nuclear factor κB kinase, MEF, mouse embryonic fibroblast, LKB1, liver kinase B1, NEM, *N*-ethylmaleimide, NUAK, NUAK family SnF1-like kinase, PEI, polyethylenimine, PI, propidium iodide, PLK1, Polo kinase 1, PP1, protein phosphatase 1, SCF^βTrCP^, Skp, Cullin and F-box^βTrCP^, SKP1, S-phase kinase-associated protein 1, Wee1, WEE1 G_2_ checkpoint kinase, WT, wild-type, XIC, extracted ion chromatogram analysis

## Abstract

NUAK1 (NUAK family SnF1-like kinase-1) and NUAK2 protein kinases are activated by the LKB1 tumour suppressor and have been implicated in regulating multiple processes such as cell survival, senescence, adhesion and polarity. In the present paper we present evidence that expression of NUAK1 is controlled by CDK (cyclin-dependent kinase), PLK (Polo kinase) and the SCF^βTrCP^ (Skp, Cullin and F-box^βTrCP^) E3 ubiquitin ligase complex. Our data indicate that CDK phosphorylates NUAK1 at Ser^445^, triggering binding to PLK, which subsequently phosphorylates NUAK1 at two conserved non-catalytic serine residues (Ser^476^ and Ser^480^). This induces binding of NUAK1 to βTrCP, the substrate-recognition subunit of the SCF^βTrCP^ E3 ligase, resulting in NUAK1 becoming ubiquitylated and degraded. We also show that NUAK1 and PLK1 are reciprocally controlled in the cell cycle. In G_2_–M-phase, when PLK1 is most active, NUAK1 levels are low and *vice versa* in S-phase, when PLK1 expression is low, NUAK1 is more highly expressed. Moreover, NUAK1 inhibitors (WZ4003 or HTH-01-015) suppress proliferation by reducing the population of cells in S-phase and mitosis, an effect that can be rescued by overexpression of a NUAK1 mutant in which Ser^476^ and Ser^480^ are mutated to alanine. Finally, previous work has suggested that NUAK1 phosphorylates and inhibits PP1β^MYPT1^ (where PP1 is protein phosphatase 1) and that a major role for the PP1β^MYPT1^ complex is to inhibit PLK1 by dephosphorylating its T-loop (Thr^210^). We demonstrate that activation of NUAK1 leads to a striking increase in phosphorylation of PLK1 at Thr^210^, an effect that is suppressed by NUAK1 inhibitors. Our data link NUAK1 to important cell-cycle signalling components (CDK, PLK and SCF^βTrCP^) and suggest that NUAK1 plays a role in stimulating S-phase, as well as PLK1 activity via its ability to regulate the PP1β^MYPT1^ phosphatase.

## Abstract 2

The present study provides insights into the biological regulation of the NUAK isoforms and highlights the remarkable interplay that exists between Polo kinase, NUAK1, PP1β^MYPT1^ and SCF^βTrCP^ signalling components. It demonstrates NUAK1 inhibitors suppress cell proliferation and PLK1.

## INTRODUCTION

NUAK1 [NUAK family SnF1-like kinase-1; also known as ARK5 (AMPK-related kinase 5)] and the closely related NUAK2 [SNARK (SNF1/AMP kinase-related kinase)] belong to the AMPK (AMP-activated protein kinase) family of protein kinases and are phosphorylated and activated by the LKB1 (liver kinase B1) tumour suppressor protein kinase [1,2]. NUAK isoforms are widely expressed and possess an N-terminal kinase domain (residues 55–306, NUAK1), followed by a non-catalytic C-terminal region (residues 307–661, NUAK1) [2]. LKB1 activates NUAK isoforms by phosphorylating the kinase domain T-loop residue (Thr^211^-NUAK1).

Previous work has indicated that NUAK isoforms play roles in a number of processes including controlling embryonic development [3,4], cell adhesion [5,6], senescence [7], neuronal polarity and axon branching [8]. Other work points towards roles of NUAK isoforms in regulating cell division, through its ability to stimulate proliferation [9], promote invasion of cancer cells [10–12] and function as a survival factor in Myc-driven tumours [13]. Despite these studies, relatively little is known about how NUAK isoforms are regulated and function. To date only a single substrate, namely the MYPT1 subunit of the PP1β^MYPT1^ (where PP1 is protein phosphatase 1) myosin phosphatase complex, has been reported, whose phosphorylation is reduced in NUAK1-knockout cells [6]. NUAK1 and NUAK2 phosphorylate MYPT1 at three conserved residues (Ser^445^, Ser^472^ and Ser^910^) in response to conditions that induce cell detachment [6]. This phosphorylation triggers binding of MYPT1 to 14-3-3 isoforms, thereby inhibiting dephosphorylation of the myosin light chain by PP1β^MYPT1^ [6]. NUAK1 and NUAK2 associate with the PP1β^MYPT1^ via a set of three highly conserved GILK motifs that bind directly to a regulatory pocket on the surface of the PP1β catalytic subunit [6].

Previous work has also revealed that PP1β^MYPT1^ acts to inactivate PLK1 by dephosphorylating the T-loop Thr^210^ residue, thereby controlling mitosis [14]. The ability of PLK1 to interact with PP1β^MYPT1^ is dependent upon phosphorylation of MYPT1 at Ser^473^ by CDK1 (cyclin-dependent kinase 1), which creates a docking site recognized by the Polo-box domains of PLK1 (Polo kinase 1) [14]. Interestingly, Ser^473^ lies immediately adjacent to the NUAK1 phosphorylation site (Ser^472^) that controls 14-3-3 binding. This therefore suggests that phosphorylation of MYPT1 by NUAK1 and 14-3-3 binding could directly interfere with the ability of PP1β^MYPT1^ to interact with and hence dephosphorylate PLK1.

In the present study we provide evidence that NUAK1 is phosphorylated by PLK at two serine residues (Ser^476^ and Ser^480^) that lie within a conserved ESGYYS phosphodegron motif located in the C-terminal non-catalytic domain of NUAK1. This phosphorylation triggers interaction with the SCF^βTrCP^ (Skp, Cullin and F-box^βTrCP^) E3 ubiquitin ligase complex, leading to polyubiquitylation and degradation of NUAK1. We demonstrate that the levels of NUAK1 are low in the G_2_–M-phase of the cell cycle when PLK1 is most active and high in the S-phase when PLK1 expression is low. We also provide pharmacological evidence that NUAK1 controls proliferation by regulating the population of cells in S-phase and mitosis. Lastly, consistent with NUAK1 inhibiting PP1β^MYPT1^ and PP1β^MYPT1^ acting on PLK1, we demonstrate that cell detachment that promotes phosphorylation and inhibition of PP1β^MYPT1^ by NUAK1 markedly enhances the T-loop phosphorylation of PLK1. The present study provides further insights into the biological regulation of the NUAK isoforms and highlights the remarkable interplay that exists between PLK, NUAK1, PP1β^MYPT1^ and SCF^βTrCP^ signalling components.

## MATERIALS AND METHODS

### Materials

[γ^32^P]ATP was from PerkinElmer. Protein G–Sepharose, glutathione–Sepharose and the ECL kit were from GE Healthcare. Sakamototide substrate peptide [ALNRTSSDSALHRRR] was used for endogenous NUAK1 activity assay as described previously [6]. P81 phosphocellulose paper was from Whatman. The CellTiter 96® AQueous Non-Radioactive Cell Proliferation Assay kit and sequencing-grade trypsin were from Promega. RNase A and paraformaldehyde 4% solution were purchased from Affymetrix. Novex 4–12% polyacrylamide Bis-Tris gels, LDS Sample Buffer, PBS/EDTA-based Cell Dissociation Buffer, hygromycin and other tissue culture reagents were from Life Technologies. Calyculin A was purchased from Cell Signalling Technology. Instant Coomassie Blue stain was from Expedeon. PEI (polyethyleneimine)was from Polysciences and 1 M magnesium acetate solution was from Fluka. Anti-HA (haemagglutinin)–agarose, anti-FLAG–agarose, DMSO, PI (propidium iodide), BSA, L-glutathione reduced, Nonidet P40, thymidine, NEM (*N*-ethylmaleimide) and benzamidine were from Sigma–Aldrich. PMSF was from Melford. The International Centre for Protein Kinase Profiling (http://www.kinase-screen.mrc.ac.uk) supplied MLN-4924, all of the small molecule protein kinase inhibitors used in the present study and purified GST (glutathione transferase) lambda phosphatase.

### Antibodies

The following antibodies were raised in sheep and affinity purified on the appropriate antigen: anti-MYPT1 [human MBP (myelin basic protein)–MYPT1 residues 714–1005, S662B, first bleed], anti-MYPT1 phospho-Ser^445^ (residues 437–452 of mouse, RLGLRKTGS*YGALAEI, S508C, first bleed), anti-MYPT1 phospho-Ser^472^ (residues 466–478 of mouse, GVMRSAS*SPRLSS, S509C, second bleed), anti-PP1β (residues 316–327 of human PP1β, TPPRTANPPKKR, S383B, third bleed), anti-LKB1 (full-length mouse LKB1, S170D, second bleed) and anti-NUAK1 (human His–NUAK1, S628B, second bleed). The commercial antibodies used in the present study were anti-βTrCP (catalogue number 4394; Cell Signaling Technology), anti-β-tubulin (catalogue number 2128; Cell Signaling Technology), anti-PLK1 (catalogue number 4513; Cell Signaling Technology), anti-PLK1 Thr^210^ (catalogue number 55840; BD Biosciences), anti-cyclin B1 (catalogue number 4135; Cell Signaling Technology), anti-(cyclin A) (catalogue number 4656; Cell Signaling Technology), anti-GAPDH (glyceraldehyde-3-phosphate dehydrogenase; catalogue number ab8245; Abcam), anti-SIK2 (salt-inducible kinase 2; catalogue number 6919; Cell Signaling Technology), anti-phosphoH3 Ser^10^ (catalogue number 3377; Cell Signaling Technology), anti-H3 histone (catalogue number 4499; Cell Signaling Technology), anti-ubiquitin (ZO458; Dako), anti-(phosphohistone H3–Alexa Fluor® 488) (catalogue number 9708; Cell Signaling Technology), anti-(FLAG peroxidase) (A8592; Sigma) and anti-(HA peroxidase) (3F10) (12013819001; Roche). HRP (horseradish peroxidase)-conjugated secondary antibodies (1:2500 dilution) were obtained from Thermo Scientific and HRP-coupled light chain-specific secondary antibodies (1:10000 dilution) were purchased from Jackson Immuno Research.

### General methods

All recombinant DNA procedures, electrophoresis, tissue culture, immunoblotting and immunoprecipitations were performed using standard protocols. All mutagenesis was performed using the QuikChange® site-directed mutagenesis method (Stratagene) with KOD polymerase (Novagen). DNA constructs were purified from *Escherichia coli* DH5α cells using QIAGEN maxi-prep kits according to the manufacturer's protocol. All DNA constructs were verified by DNA sequencing, which was performed by the Sequencing Service (MRC Protein Phosphorylation Unit, College of Life Sciences, University of Dundee, Dundee, U.K.; http://www.dnaseq.co.uk), using DYEnamic ET terminator chemistry (GE Healthcare) on Applied Biosystems automated DNA sequencers. Cell proliferation assay was carried out using the CellTiter 96® AQueous Non-Radioactive Cell Proliferation Assay kit as described previously [15].

### Cell culture, treatments and cell lysis

U2OS and HEK (human embryonic kidney)-293 cells were cultured in DMEM (Dulbecco's modified Eagle's medium) supplemented with 10% FBS, 2 mM glutamine and 1× antibacterial/antimycotic solution. βTrCP1^+/+^ and βTrCP1^−/−^ MEFs (mouse embryonic fibroblasts) were kindly provided by Professor Keiichi Nakayama (Kyushu University, Fukuoka, Japan) and were cultured in DMEM supplemented with 10% (v/v) FBS, 2 mM glutamine, 1× antibacterial/antimycotic solution, 1% (v/v) non-essential amino acids and 1% (v/v) sodium pyruvate. Transient transfections of HEK-293 cells were carried out using PEI. U2OS Flp/In cells were kindly provided by Professor John Rouse (University of Dundee, Dundee, U.K.) and stable transfections were carried out in the cells following a standard protocol (Invitrogen). Post stable transfection, the U2OS Flp/In cells were selected and cultured in DMEM supplemented with 10% (v/v) FBS, 2 mM glutamine, 1× antibacterial/antimycotic solution and 100 μg/ml hygromycin. Inhibitor treatments were carried out by treating the cells with various concentrations of the inhibitors as indicated in the Figure legends. The inhibitors were dissolved in DMSO and the total concentration of DMSO in the culture medium never exceeded 1%. Cells were lysed in lysis buffer containing 50 mM Tris/HCl (pH 7.5), 1 mM EGTA, 1 mM EDTA, 1% Triton X-100, 50 mM NaF, 10 mM sodium 2-glycerophosphate, 5 mM sodium pyrophosphate, 1 mM sodium orthovanadate, 0.27 M sucrose, 1 mM benzamidine (added before lysis), 1 mM PMSF (added before lysis) and 0.1% 2-mercaptoethanol (added before lysis). To observe ubiquitylation in immunoblotting, cells were lysed in lysis buffer containing 20 mM NEM minus any reducing agent. Lysates were clarified by centrifugation at 16000 ***g*** for 15 min at 4°C and either used for further experiments or snap frozen in liquid nitrogen and stored at −80°C. Protein estimation was carried out using Bradford method with BSA as a standard.

### Lambda phosphatase assay

Endogenous NUAK1 was immunoprecipitated from 20 mg of U2OS cells treated with 50 nM calyculin A. NUAK1 immunoprecipitates were incubated with either 10 μg of active GST-lambda phosphatase or 50 mM EDTA-inactivated 10 μg of GST-lambda phosphatase in a reaction volume of 50 μl consisting of 50 mM Tris/HCl (pH 7.5), 1 mM MnCl_2_ and 0.1% 2-mercaptoethanol. Assays were incubated at 30°C for 30 min. The beads were washed three times in 50 mM Tris/HCl (pH 7.5), 0.1 mM EGTA and 0.5 M NaCl followed by washing two times in 50 mM Tris/HCl (pH 7.5) and 0.1 mM EGTA. Samples were analysed by immunoblotting.

### Identification of NUAK1-interacting proteins by MS and development of extracted ion chromatogram for phosphopeptide

U2OS Flp/In empty (control) or with overexpression of HA–NUAK1 were lysed and HA–NUAK1 was immunoprecipitated from 35 mg of lysate. Proteomic mass fingerprint analysis was carried out to identify potential interactors of NUAK1 as described previously [6]. Results were searched against the SwissProt or IPI human database using Mascot (http://www.matrixscience.com). Peptide mass fingerprinting data analysis was performed using OLMAT (http://www.proteinguru.com/MassSpec/OLMAT).

HA–NUAK1, with or without 50 nM calyculin A, and HA–NUAK1 S476A+S480A were immunoprecipitated from U2OS Flp/In cells expressing either the WT (wild-type) or the mutant HA–NUAK1. The immunoprecipitates were resolved on a polyacrylamide gel that was stained with Coomassie Blue. Bands migrating with the expected molecular mass of HA–NUAK1 were excised from the gel, digested with trypsin and subjected to HPLC-MS/MS on an ABSciex QTrap 4000 mass spectrometer using precursor ion scanning for −79 Da [16]. XIC (extracted ion chromatogram analysis; where the total signal intensity of the phosphopeptide was plotted on the *y*-axis and retention time was plotted on the *x*-axis) of the Ser^476^- and Ser^480^-containing phosphopeptide (R.ESGYYSSPER.S+1P) was obtained manually using Analyst software (ABSciex). Similarly, HA–NUAK1, with or without 1 μM BI2536 treatment, was analysed by LC-MS/MS on a Thermo LTQ-Orbitrap and XIC data was obtained manually using Xcalibur software (Thermo).

### Kinase assay

Endogenous NUAK1 was immunoprecipitated from 5 mg of βTrCP1^+/+^ and βTrCP1^−/−^ MEFs in triplicates. Radioactive kinase assay was carried out using Sakamototide substrate peptide and [γ^32^P]ATP-Mg^2+^ as described previously [6]. The samples were further subjected to immunoblotting to visualize the levels of NUAK1.

### Cell cycle synchronization

U2OS cells were synchronized in the cell cycle by either DTB (double thymidine block)–release for G_1_–S or by DTB with a RO-3306 release for G_2_–M–G_1_. U2OS cells were plated at 40% confluency and treated with 2.5 mM thymidine for 16 h. Cells were subsequently washed twice with PBS and fresh medium was added and cells were released from block for 12 h. A second 2.5 mM thymidine block was introduced for another 16 h. Following the DTB, cells were washed three times with PBS, fresh media was added, and cells were harvested for flow cytometry and immunoblotting at various time points as mentioned in the Figure legends. For G_2_–M–G_1_ synchronization, after the DTB, cells were washed three times in PBS and released in fresh medium for 6 h. After release, 10 μM RO-3306 was added to the cells for 20 h. After DTB+RO-3306 treatment, cells were washed three times in PBS, fresh medium was added, and cells were harvested for flow cytometry and immunoblotting at various time points as mentioned in the Figure legends.

### Analysis of cell cycle by flow cytometry

U2OS cells were analysed for their respective cell cycle phase distribution using flow cytometry. Post-inhibitor treatment, cells were trypsinized, washed with PBS+0.2% BSA and resuspended in flow cytometry tubes. Cells were then fixed by 70% ice-cold ethanol and stored at −20°C until analysis. After washing fixed cells once with PBS, RNase A (50 μg/ml) and PI (50 μg/ml) were added to the cells and incubated in the dark at room temperature (25°C) for 20 min. The live cell populations were then subjected to quantitative measurement of DNA content by flow cytometry using a FACSCalibur™ (BD Biosciences) and cell cycle distribution and the percentage of G_2_–S–G_1_ cells determined by the Watson (pragmatic) modelling algorithm using FlowJo software (Treestar).

### Calculation of mitotic cells by flow cytometry

U2OS cells were analysed for their respective percentage of mitotic cells using flow cytometry. The cell medium was collected, centrifuged and the floating population of mitotic cells were collected in flow cytometry tubes. The adherent cells were try-psinized, washed with PBS+0.2% BSA and resuspended in the same flow cytometry tubes. Cells were then resuspended in 0.5 ml of 1% paraformaldehyde in PBS and incubated at 37°C for 15 min. Cells were washed with PBS/BSA and then fixed by 90% ice-cold methanol and stored at −20°C until analysis. After washing fixed cells once with PBS, the cells were resuspended in 500 μl of diluted anti-(phosphohistone H3–Alexa Fluor® 488) (1:200 dilution) in PBS. Cells were incubated for 1 h at room temperature. Cells were washed in PBS/BSA and resuspended in staining buffer containing RNase A (50 μg/ml) and PI (50 μg/ml) and incubated in the dark at room temperature for 20 min. The cells were then subjected to quantitative measurement of DNA content and Alexa Fluor® 488 intensity by flow cytometry using a FACS Calibur (BD Biosciences). The percentage of mitotic cells determined by the Watson (pragmatic) modelling algorithm using FlowJo software (Treestar).

### Calculation of mitotic cells by microscopy

U2OS cells were split and approximately equal number of cells was loaded into a 12-well plate. To study the effect of NUAK1 inhibition for mitosis, bright-field imaging was carried out with or without treatment with DMSO (control) or 10 μM of WZ4003, 3 μM HTH-01-015 or 10 μM of RO-3306 (control of mitotic defect). Inhibitors were added to the cells before the start of the imaging. The imaging was carried out under a Nikon Eclipse Ti microscope using objective ×20 phase with images taken every 1 min by a Photometrics cascade II CCD (charge-coupled device) camera using Nikon NIS Elements software. All drug treatments were collected under the same conditions using the multi-point visiting stage facility. The experiments were carried out in triplicate. The percentage of cells in the field forming mitotic contractile rings over a period of 1000 min was manually counted from three independent fields of view and the data were plotted using GraphPad Prism software. Only those cells forming mitotic contractile rings were included, i.e. those which eventually completed successful mitosis and formed two daughter cells.

### Statistical analysis

All experiments described in the present paper were performed at least twice and similar results were obtained. Data were analysed using Student's *t* test or one-way ANOVA followed by multiple pair-wise comparisons (*P<0.05). Error bars indicate S.D.

## RESULTS

### Interaction of NUAK1 with components of the SCF^βTrCP^ E3 ligase complex

**Figure 1:**
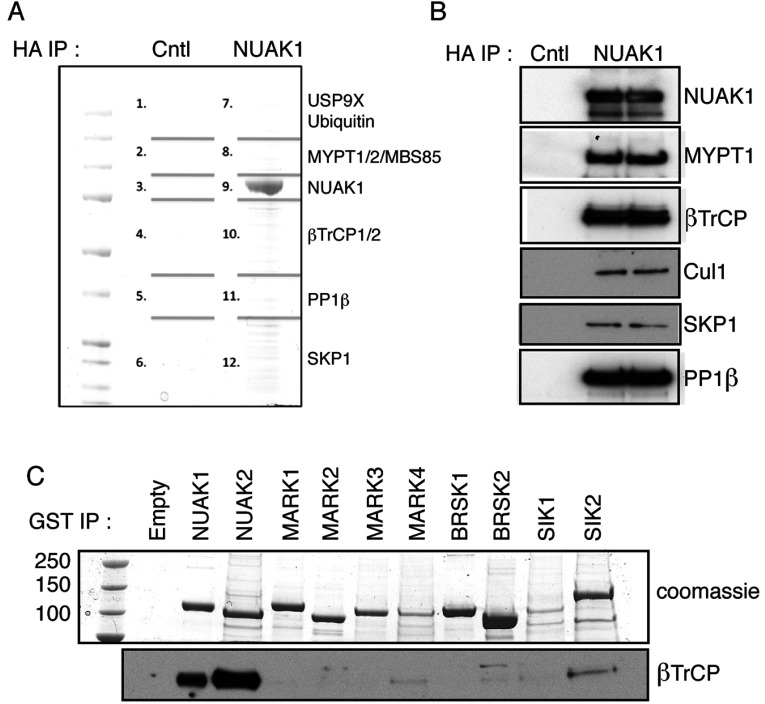
NUAK1 binds to βTrCP (**A**) HA–NUAK1 was immunoprecipitated (IP) from U2OS Flp/In cells expressing empty vector (Cntl) or with overexpression of HA–NUAK1. The immunoprecipitates were resolved on a polyacrylamide gel and was stained with Coomassic Blue. The gel was divided into the indicated pieces, and proteins in these pieces were identified by mass spectrometry. Previously published interactors ubiquitin and USP9X were identified in band 7, MYPT1, MYPT2 and MBS85 were identified in band 8, PP1β in band 11, and NUAK1 in band 9. βTrCP1 and βTrCP2 was identified in band 10, whereas SKP1 was identified in band 12. Mascot scores of the interactors are detailed in Supplementary Table S1 (http://www.biochemj.org/bj/461/bj4610233add.htm). (**B**) Overexpressed HA–NUAK1 was immunoprecipitated from U2OS Flp/In cell lysate stably overexpressing HA–NUAK1 and subjected to immunoblotting with the indicated antibodies. U2OS Flp/In cell lysate expressing empty vector was used as a negative control. (**C**) HEK-293 cells were transfected with expression plasmids for GST-tagged AMPK-related kinases. At 36 h after transfection cells were lysed and GST-tagged proteins were immunoprecipitated from 5 mg of cell lysates. Immunoprecipitates were analysed by immunoblotting with indicated antibodies. MARK, MAP/microtubule affinity-regulating kinase.

We employed mass spectrometry to identify proteins that specifically immunoprecipitated with stably overexpressed NUAK1 in osteosarcoma-derived U2OS cells (Fig-ure 1A). NUAK1 was expressed ~20-fold higher than endogenous NUAK1 in these experiments (Supplementary Figure S1 at http://www.biochemj.org/bj/461/bj4610233add.htm). These studies revealed that in addition to previously reported NUAK1 interactors USP9X [17] and PP1β^MYPT1^ complex components [6], we also observed constituents of the SCF^βTrCP^ E3 ubiquitin ligase complex (Figures 1A and 1B, and Supplementary Table S1 at http://www.biochemj.org/bj/461/bj4610233add.htm). Both closely related βTrCP1 and βTrCP2 isoforms that function as the F-box-containing substrate-recognition components of the SCF E3 ubiquitin ligase complexes [18,19], as well as SKP1 (S-phase kinase-associated protein 1) and Cul1 (cullin 1) subunits, were associated with NUAK1 immunoprecipitates (Figures 1A and 1B). βTrCP co-purified with overexpressed NUAK1 and NUAK2, but not with the eight other AMPK-related protein kinases tested (Figure 1C).

### Phosphorylation of NUAK1 at Ser^476^ and Ser^480^ control association with βTrCP

Previous work has demonstrated that βTrCP isoforms possess multiple WD40 motifs at their C-terminus, which fold into a target-recognizing phosphate-binding pocket [20]. βTrCP binds to the target proteins after they are phosphorylated at two residues located four amino acids apart within a phosphodegron motif. Different target proteins are phosphorylated by different kinases, e.g. IκB (inhibitor of nuclear factor κB) by IKKs (IκB kinases) [21], β-catenin by GSK3β (glycogen synthase kinase 3β) and CK1 (casein kinase 1) [22], and Wee1 (WEE1 G_2_ checkpoint kinase) by PLK [23]. These phosphorylations recruit the SCF^βTrCP^ E3 ubiquitin ligase, resulting in the target becoming ubiquitylated and degraded by the proteasome.

**Figure 2:**
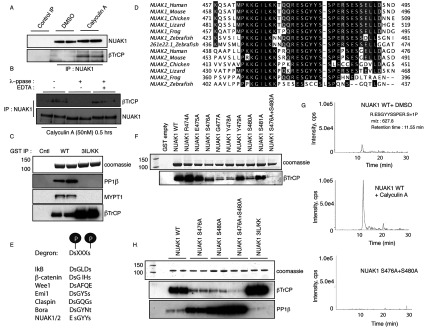
NUAK1 degron requires phosphorylation to interact with βTrCP (**A**) U2OS cells were treated with or without 50 nM calyculin A for 10 min and the cells were lysed and endogenous NUAK1 was immunoprecipitated (IP) from 15 mg of cell lysates. Immunoprecipitates were analysed by immunoblotting with indicated antibodies. Pre-immune IgG was used as a control. (**B**) Endogenous NUAK1 was immunoprecipitated from U2OS cells treated with 50 nM calyculin A for 30 min. The immunoprecipitates were subjected to lambda-phosphatase assay with or without prior inactivation of lambda-phosphatase with EDTA. The immunoprecipitates were washed thoroughly and were analysed by immunoblotting with indicated antibodies. (**C**) HEK-293 cells were transfected with expression plasmids for the GST-tagged NUAK1 WT or NUAK1 3IL/KK mutant. At 36 h post-transfection cells were lysed and GST-tagged proteins were immunoprecipitated from 5 mg of cell lysates. Immunoprecipitates (IP) were analysed by immunoblotting with the indicated antibodies. (**D**) Alignment showing the conservation of the ESGYYS degron of NUAK1 and NUAK2 within vertebrate orthologues. The sequences of *Homo sapiens*, *Mus musculus*, *Gallus gallus*, *Xenopus tropicalisi*, *Anolis carolinensis* and *Danio rerio* NUAK1 and NUAK2 are from the NCBI. There are two copies of NUAK1 in *D. rerio*: one on chr. 4 and another on chr. 25. Alignment was performed using T-Coffee software and visualized using Jalview. (**E**) Alignment of the candidate phosphodegron sequence DSGxxS between NUAK1 and the canonical βTrCP-interacting target proteins IkB, β-catenin, Wee1, Emi1, claspin and Bora. (**F**) HEK-293 cells were transfected with expression plasmids for the GST-tagged NUAK1 WT or indicated mutants and immunoprecipitations and immunoblottings were carried out as in (**C**). (**G**) HA–NUAK1 with or without calyculin A treatment and HA–NUAK1 S476A+S480A were immunoprecipitated (IP) from U2OS Flp/In cells expressing either the WT or the mutant HA–NUAK1. The immunoprecipitates were resolved on a polyacrylamide gel and stained with Coomassie Blue. After trypsin digestion and precursor ion scanning (−79 Da) on the QTrap 4000 an XIC analysis of the Ser^476^ and Ser^480^ containing phosphopeptide (R.ESGYYSSPER.S+1P) was carried out. The *m*/*z* value corresponding to this phosphopeptide was detected in NUAK1 WT (both DMSO- and calyculin A-treated), but not in the S476A+Ser480A mutant. In the calyculin A-treated cells, the *m*/*z* value corresponding to the Ser^476^- or Ser^480^ -containing phosphopeptide was found to possess a >6-fold higher signal intensity than DMSO treated (control) immunoprecipitate. (**H**) HEK-293 cells were transfected with expression plasmids for the GST-tagged NUAK1 WT or indicated mutants and immunoprecipitations and immunoblotting was carried out as in (**C**).

To investigate whether interaction between βTrCP isoforms and NUAK1 is controlled by phosphorylation, we first treated U2OS cells in the presence or absence of the protein phosphatase inhibitor calyculin A [24] to see whether this enhances phosphorylation of NUAK1 and hence binding to βTrCP. This revealed that calyculin A within 30 min markedly enhanced association of βTrCP isoforms to endogenous NUAK1 (Figure 2A). Consistent with the association of βTrCP with NUAK1 being mediated through phosphorylation, treatment of endogenous NUAK1 immunoprecipitates with lambda phosphatase-induced dissociation of βTrCP isoforms (Figure 2B).

As mentioned in the Introduction section, NUAK1 interacts with the PP1β^MYPT1^ myosin phosphatase [6]. To investigate whether disruption of the binding of PP1β^MYPT1^ to NUAK1 might also promote NUAK1 phosphorylation and hence interaction with SCF^βTrCP^, we mutated the three GILK motifs in NUAK1 that mediate PP1β^MYPT1^ phosphatase binding [6] and observed that this significantly enhanced binding of βTrCP (Figure 2C).

To identify the residues on NUAK1 that mediate binding to βTrCP, we employed mass spectrometry to map phosphoryla-tion sites on WT NUAK1. We also mapped phosphorylation sites on the non-PP1β^MYPT1^-binding NUAK1 mutant, as the results presented in Figure 2(C) indicate that the βTrCP-binding phosphorylation residues should be more highly phosphorylated in this mutant than the WT. We therefore searched for NUAK1 phosphorylation sites located four residues apart that might constitute a βTrCP-binding phosphodegron motif, which were enhanced by ablation of the PP1β^MYPT1^-binding GILK motifs (Supplementary Figure S2 at http://www.biochemj.org/bj/461/bj4610233add.htm). From this analysis, a doubly phosphorylated peptide meeting these criteria was identified, encompassing Ser^476^ and Ser^480^, that the mass spectrometry data suggested comprised the sites of phosphorylation. These residues also lie within an ESGYYS motif (residues corresponding to Ser^476^ and Ser^480^ are underlined), which is highly conserved between NUAK isoforms in all of the species we have analysed (Figure 2D) and similar to most other characterized βTrCP-binding phosphodegron motifs (Figure 2E) [25].

Consistent with phosphorylation of Ser^476^ and Ser^480^ mediating βTrCP binding, mutation of these residues to alanine abo-lished βTrCP isoform binding to NUAK1 (Figure 2F). Binding to βTrCP was also suppressed by mutation of the conserved adjacent Gly^477^ and Tyr^478^ (Figures 2E and 2F). We also observed using a quantitative mass spectrometry approach that 50 nM calyculin A enhanced phosphorylation of Ser^476^ and Ser^480^ approximately 6-fold (Figure 2G), which is likely to account for increased association of βTrCP with NUAK1 under these conditions (Figure 2A).

Ser^476^ and Ser^480^ lie adjacent to one of the conserved PP1β^MYPT1^-binding GILK motif 2 (residues 466–469), indicating the potential for competition between βTrCP and PP1β^MYPT1^ binding to NUAK1 (Figure 2D). We observed that mutation of Ser^476^ and Ser^480^ to alanine to ablate βTrCP binding markedly enhanced association of NUAK1 to PP1β^MYPT1^ (Figure 2H). Mutation of the GILK motif 2 (residues 466–469), that lies nearby Ser^476^ and Ser^480^, had no impact on βTrCP binding, but mutation of motif 1 (residues 399–402) or motif 3 (residues 523–526) or all three GILK motifs (3IL/KK) enhanced βTrCP binding (Figure 2H, and Supplementary Figure S3 at http://www.biochemj.org/bj/461/bj4610233add.htm).

### Evidence that NUAK1 is targeted for degradation by the SCF^βTrCP^ E3 ligase complex

**Figure 3:**
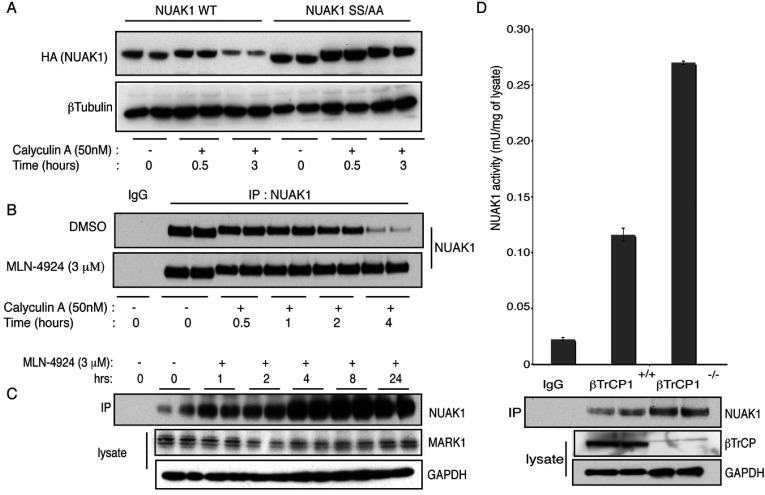
NUAK1 is protected from phosphorylation-mediated degradation upon SCF^βTrCP^ inhibition (**A**) U2OS cells stably expressing NUAK1 WT or S476A+S480A mutant were treated with 50 nM calyculin A over the indicated periods of time. The cell lysates were analysed by immunoblotting with indicated antibodies. (**B**) Endogenous NUAK1 was immunoprecipitated (IP) from 1 mg of U2OS cell lysates treated with calyculin A (50 nM) and with or without MLN-4924 (3 μM) over the indicated periods of time prior to lysis. Immunoblotting was carried out to detect NUAK1 levels in the immunoprecipitates. Pre-immune IgG was used as a control. (**C**) Endogenous NUAK1 was immunoprecipitated from 1 mg of U2OS cell lysates treated with MLN-4924 (3 μM) over the indicated periods of time prior to lysis. Immunoprecipitates were analysed by immunoblotting with indicated antibodies. (**D**) βTrCP1^+/+^ (WT) and βTrCP1^−/−^ (knockout) MEFs were lysed and analysed by immunoblotting with the indicated antibodies. Endogenous NUAK1 was immunoprecipitated and its activity was assayed in triplicates with pre-immune IgG as control. Results are means±S.D.

To investigate whether the expression of NUAK1 was controlled by the SCF^βTrCP^ E3 ligase complex, we first treated U2OS cells stably expressing WT NUAK1 with 50 nM calyculin A to induce βTrCP binding. This revealed that following 3 h of calyculin A treatment, the levels of WT NUAK1, but not the non-βTrCP-binding NUAK1[S476A+S480A] mutant, were markedly reduced (Figure 3A).

Treating cells with calyculin A to enhance NUAK1 phosphorylation induced significant degradation of endogenous NUAK1 within a 2–4 h time frame (Figure 3B). This effect of calyculin A was blocked by treating cells with the specific MLN4924 inhibitor that inactivates SCF E3 ubiquitin ligase complexes by suppressing NEDDylation of Cul-1 [26] (Figure 3B). Treatments of cells with MLN-4924 resulted in significant increase in the expression of endogenous NUAK1 at the 4–24 h time points (Figure 3C). In contrast, MLN-4924 had no effect on the levels of the MARK1 (MAP/microtubule affinity-regulating kinase 1), an AMPK-related kinase (Figure 3C) that does not interact with βTrCP (Figure 1C). Both endogenous and overexpressed NUAK1 underwent significant polyubiquitylation upon calyculin A treatment, which was reversed upon pre-treating the cells with MLN-4924 (Supplementary Figure S4 at http://www.biochemj.org/bj/461/bj4610233add.htm).

To obtain further evidence that βTrCP1 regulates NUAK1, we investigated how knockout of the βTrCP1 isoform in previously described MEF cells [27] impacted on endogenous NUAK1 expression. These experiments revealed that NUAK1 was expressed at a ~2-fold-higher expression in βTrCP1-knockout cells (that still express βTrCP2) (Figure 3D). Moreover, endogenous NUAK1 catalytic activity assessed after an immunoprecipitation protein kinase activity assay, was also enhanced ~2-fold in βTrCP1-knockout cells (Figure 3D).

### Evidence that Polo kinase phosphorylates Ser^476^ and Ser^480^

**Figure 4:**
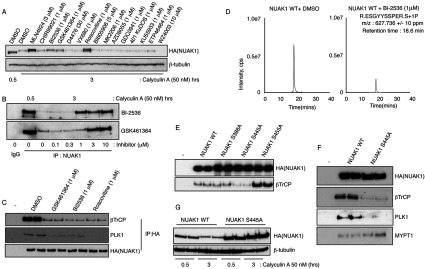
PLK1 interacts with NUAK1 at the CDK priming Ser^445^ site and phosphorylates NUAK1 at Ser^476^ and Ser^480^ and targets it for phosphorylation-mediated degradation (**A**) U2OS cells stably expressing HA–NUAK1 were treated with the indicated inhibitors for 4 h with or without 50 nM calyculin A treatment. Cell lysates were subjected to immunoblotting with the indicated antibodies. The inhibitors were used at 1 μM final except for MLN-4924 (3 μM), BI605906 (5 μM), WZ4003 (10 μM) and D4476 (30 μM). (**B**) Endogenous NUAK1 was immunoprecipitated (IP) from 1 mg of U2OS cell lysates treated with calyculin A (50 nM) and with or without the indicated concentrations of BI2536 and GSK461364 over the indicated periods of time prior to lysis. Immunoblotting was carried out to detect NUAK1 levels in the immunoprecipitates. Pre-immune IgG was used as a control. (**C**) U2OS cells stably expressing NUAK1 WT were treated with the indicated inhibitors for 1 h. HA–NUAK1 was immunoprecipitated and analysed by immunoblotting with the indicated antibodies. (**D**) HA–NUAK1 with or without BI2536 (1 μM) treatment was immunoprecipitated (IP) from U2OS Flp/In cells stably expressing HA–NUAK. XIC analysis of the Ser^476^- and Ser^480^-containing phosphopeptide (R.ESGYYSSPER.S+1P) was carried out. The *m*/*z* value corresponding to the Ser^476^- or Ser^480^-containing phosphopeptide was detected in both samples. In the BI253-treated cells, the *m*/*z* value corresponding to the Ser^476^- or Ser^480^-containing phosphopeptide was found to possess a ~3-fold-higher signal intensity than the control immunoprecipitate. (**E**) HEK-293 cells were transfected with expression plasmids for the HA-tagged NUAK1 WT or the indicated mutants. At 36 h post-transfection cells were lysed and HA-tagged proteins were immunoprecipitated from 1 mg of cell lysates. Immunoprecipitates (IP) were analysed by immunoblotting with indicated antibodies. (**F**) HEK-293 cells were transfected with expression plasmids for the HA-tagged NUAK1 WT or S445A mutant. At 36 h post-transfection cells were lysed and HA-tagged proteins were immunoprecipitated from 1 mg of cell lysates. Immunoprecipitates (IP) were analysed by immunoblotting with indicated antibodies. (**G**) HEK-293 cells transiently overexpressing NUAK1 WT or the S445A mutant were treated with 50 nM calyculin A over the indicated periods of time. The cell lysates were analysed by immunoblotting with the indicated antibodies.

In an attempt to pinpoint the kinase that phosphorylates NUAK1 at Ser^476^ and Ser^480^, we investigated the effect of a variety of selective protein kinase inhibitors known to target kinases that phosphorylate βTrCP-binding phosphodegron motifs on other targets. These included inhibitors of IKKs (BI605906), GSK3β (CHIR99021), polo-kinases (BI2536 and GSK461364), CK1 (D4476), aurora kinase (VX680) and CDKs (roscovitine). We also tested a number of selective inhibitors of major protein kinase signalling systems namely ATM (ataxia telangiectasia mutated; KU55933), DNA-PK (DNA-dependent protein kinase, catalytic subunit; 401KuDOS), ATR (ataxia telangiectasia and Rad3 related; ETP46464), mTOR (mammalian target of rapamycin; AZD8055), PI3K (phosphoinositide 3-kinase; GDC0941) and Akt (MK2206). This analysis strikingly revealed that two structurally unrelated inhibitors of polo kinases (PLK1, PLK2 and PLK3), namely BI2536 [28] or GSK461364 [29], prevented the calyculin A-mediated decrease in the levels of overexpressed WT NUAK1 (Figure 4A). BI2536 (http://www.kinase-screen.mrc.ac.uk/screening-compounds/341035) and GSK461364 (http://www.kinase-screen.mrc.ac.uk/screening-compounds/224) are highly potent and specific inhibitors of PLK1, PLK2 and PLK3, but not PLK4 [30]. Dose-dependence analysis indicated that the lowest dose of BI2536 and GSK461364 that inhibited the degradation of NUAK1 was 1 μM and 3 μM respectively (Figure 4B), in accordance with the reported cellular IC_50_ of these compounds [28,29]. Consistent with PLK mediating phosphorylation of Ser^476^ and Ser^480^, we found that BI2536 and GSK461364 inhibited binding of βTrCP to overexpressed NUAK1 (Figure 4C). Mass spectrometry analysis also revealed that treatment of U2OS cells with BI2536 inhibitor significantly suppressed phosphorylation of NUAK1 at Ser^476^ and Ser^480^ (Figure 4D).

### Evidence that CDK phosphorylates NUAK1 at Ser^445^ to enable docking of PLK

In addition to the PLK inhibitors, we found that the CDK inhibitor roscovitine stabilized NUAK1 expression following calyculin A treatment (Figure 4A). Many reported PLK1 substrates, such as aurora A kinase [31,32] and the Wee1 kinase regulator of mitotic entry [23], are primed for PLK1 phosphorylation by prior phosphorylation at a serine–proline motifs by CDKs. This creates a docking site recognized by a pair of conserved polo-box regions of 30 amino acids at the C-terminus that operate as substrate-recognition domains within the C-terminal domain of PLK1 [33,34]. The finding that roscovitine inhibited NUAK1 degradation following calyculin A treatment suggests that the ability of PLKs to phosphorylate Ser^476^/Ser^480^ is dependent upon prior phosphorylation of NUAK1 by CDKs. Consistent with this, PLK1 that co-immunoprecipitates with NUAK1 is dissociated, along with βTrCP, upon treatment of cells with roscovitine (Figure 4C).

To determine the potential CDK phosphorylation site(s) on NUAK1 that induce PLK binding, we inspected the NUAK1 phosphorylation sites we have mapped (Supplementary Figure S2) for potential phosphorylated residues lying within serine/threonine–proline motifs. This revealed three sites meeting this criteria namely Ser^388^, Ser^445^ and Ser^455^ (Supplementary Figure S2). Ser^388^ [35,36], Ser^445^ [37] and Ser^455^ [36,37] are located within the C-terminal non-catalytic residues of NUAK1 and phosphorylation of these sites have also been observed in previous global phosphoproteomic studies. We also observed that phosphorylation of these residues is increased in the non-PP1β^MYPT1^ NUAK1 mutant (Supplementary Figure S2). Ser^388^ and Ser^445^, as well as the adjacent +1 proline, are conserved in all of the species we have analysed. In contrast, the +1 proline residue next to Ser^455^ in human NUAK1 is not conserved in many species including mice and rats. Our data suggest that Ser^445^ comprises the critical CDK phosphorylation site controlling PLK1 binding as mutation of Ser^445^, but not Ser^388^ or Ser^455^, suppressed interaction of NUAK1 with βTrCP (Figure 4E) as well as PLK1 (Figure 4F). Furthermore, the NUAK1[S445A] mutant was not degraded following treatment of cells with 50 nM calyculin A for 3 h (Figure 4G).

### Evidence that PLK controls NUAK1 expression in the cell cycle

**Figure 5:**
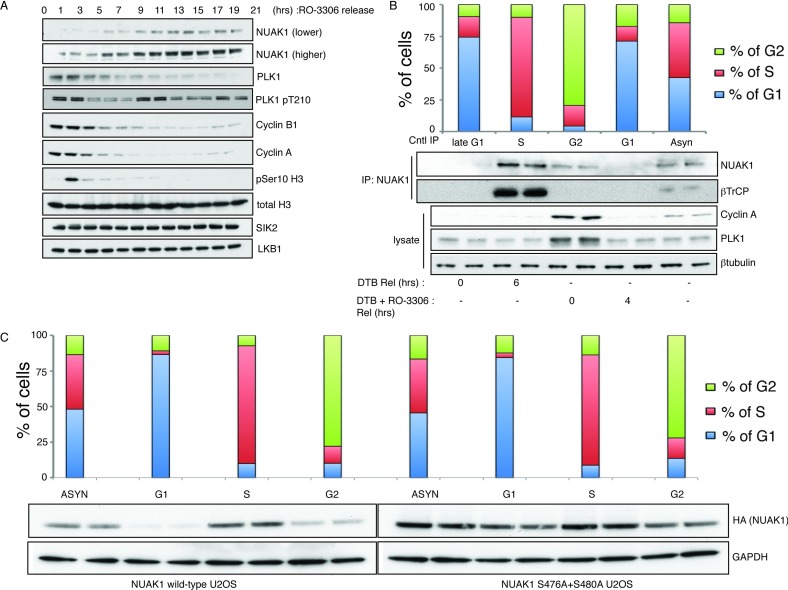
NUAK1 protein levels are controlled by SCF^βTrCP^ and PLK in the cell cycle (**A**) U2OS cells were synchronized in the G_2_ stage of the cell cycle by a DTB and release after 10 μM RO-3306 inhibitor treatment. Cells were lysed after every 2 h over 24 h. Lysates were subjected to immunoblotting with the indicated antibodies. (**B**) U2OS cells were synchronized in either in the G_1_/S stage of the cell cycle by a DTB or in the G_2_ stage by a DTB and release (Rel) after 10 μM RO-3306 inhibitor treatment. Cells were either collected or lysed after indicated times. Collected cells were subjected to quantitative measurement of DNA content (PI staining) by flow cytometry using a FACSCalibur™ (BD Biosciences) and the percentage of G_1_–S–G_2_ cells were determined by the Watson (pragmatic) modelling algorithm using FlowJo software (Treestar). Endogenous NUAK1 was immunoprecipitated from 30 mg of cell lysates and immunoblotting was carried out using the indicated antibodies. (**C**) U2OS Flp/In cells stably expressing HA–NUAK1 WT or S476A+S480A were synchronized as in (**B**) and the cell lysates were subjected to immunoblotting using the indicated antibodies.

PLK1 activity peaks at late S- and G_2_–M-phase of the cell cycle before declining in the G_1_- to early S-phase [38]. To investigate whether PLK1 regulates NUAK1 expression in the cell cycle, we synchronized U2OS cells in the G_2_ stage of the cell cycle using the DTB and CDK1 inhibitor (RO3306) release protocol [39]. Cells were then lysed at intervals over a 21-h period and immunoblotted for NUAK1, PLK1 and other cell cycle control markers (cyclin B1, cyclin A and phosphohistone H3 Ser^10^). Consistent with a role of PLK1 in targeting NUAK1 for degradation, we observed low levels of NUAK1 during the G_2_–M-phase (0–1 h time point), when PLK1 as well as cyclin A and B1 were elevated (Figure 5A). Expression levels of NUAK1 remained low in early G_1_-phase (3–5 h), increased at the 5–7 h time points and were maximal from the 9 h time point onwards (early S-phase to asynchronous) when PLK1 levels are low.

We also studied whether association of βTrCP with NUAK1 in G_1_-, S-, G_2_- and early G_1_-phase of the cell cycle. Synchronization efficiency in these studies was ascertained using flow cytometry and immunoblotting for cyclin A and PLK1 (Figure 5B). This revealed that levels of NUAK1 and co-immunoprecipitating βTrCP were highest at the S-phase of the cycle when PLK1 levels are low (Figure 5B). Levels of NUAK1 and co-immunoprecipitating βTrCP were very low at G_2_-phase as well as early and late G_1_-phase (Figure 5B). Although expression of PLK1 is low in S-phase, it has been shown to be very active especially during DNA replication [40,41]. We also observed high PLK1 activity during S-phase as indicated by its Thr^210^ phosphorylation (Figure 5A), which is likely to explain why interaction of NUAK1 with βTrCP is maximal at S-phase. It should also be noted that our data do not rule out the possibility of PLK2 or PLK3 isoforms that we have not investigated could also play a role in phosphorylating NUAK1 at the S/G_2_-phase.

We also observed that ablation of Ser^476^ and Ser^480^ markedly inhibited the reduction in NUAK1 levels observed at the G_1_- and G_2_-phases in parallel studies (Figure 5C). We reproducibly observed that expression of the NUAK1[S476A+S480A] mutant was still moderately lowered at G_1_- and G_2_-phases (Figure 5C), suggesting that an alternative mechanism not involving phosphorylation of these residues may operate to lower NUAK1 at these phases of the cell cycle. Sequence inspection indicates that NUAK1 possesses a canonical KEN box motif (KENDFAQ residues 373–379), which might trigger binding and consequent Lys^11^-linked ubiquitylation and degradation via the E3 ligase APC/C (anaphase-promoting complex/cyclosome) in the cell cycle [42,43].

### Evidence that NUAK1 promotes cell proliferation by triggering mitosis

**Figure 6:**
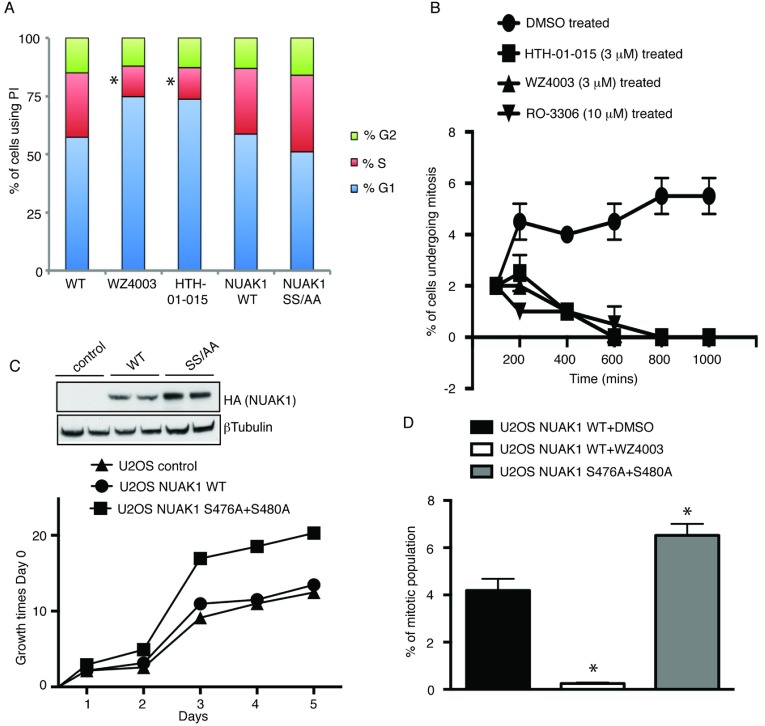
NUAK1 degradation is required for controlled mitotic progression (**A**) U2OS cells treated with or without 10 μM of WZ4003 or 3 μM HTH-01015 and U2OS Flp/In cells stably expressing NUAK1 WT and NUAK1 S476A+S480A were fixed in ice-cold 70% ethanol, stained with PI and analysed for cell cycle distribution by flow cytometry (as described in the Materials and methods section). The percentage of S-phase cells were calculated between control and inhibitor treated using GraphPad Prism. *P<0.05. (**B**) U2OS cells were treated with or without DMSO (control) or 10 μM of WZ4003 or 3 μM HTH-01015 or 10 μM of RO-3306 (control of mitotic defect). Mitotic cells were manually counted and plotted using GraphPad Prism software as elaborated in the Materials and methods section. (**C**) NUAK1 levels in U2OS Flp/In NUAK1 WT and S476A+S480A (SS/AA) were compared using immunoblotting of the cell lysates. Proliferation rate between U2OS Flp/In NUAK1 WT and S476A+S480A (SS/AA) was compared over 5 days using CellTiter 96® AQueous Non-Radioactive Cell Proliferation Assay kit. Data were represented as percentage of growth times the Day 0 counts. (**D**) U2OS Flp/In NUAK1 WT (with or without 10 μM of WZ4003 treatment) and S476A+S480A (SS/AA) cells were harvested by centrifugation, fixed in 1% paraformaldehyde, permeabilized with 90% ice-cold methanol and stained with anti-phosphohistone H3–Alexa Fluor® 488 antibody to quantify the mitotic population. The data are represented as the percentage of mitotic population and graphs were developed using GraphPad Prism. Results are means±S.D. *P<0.05.

We next investigated the effect that inhibiting NUAK1 catalytic activity had on the cell cycle by treating asynchronous U2OS cells for 8 h with two structurally distinct and highly selective NUAK1 inhibitors termed WZ4003 and HTH-01-015 [15]. This revealed that both WZ4003 and HTH-01-015, under conditions which they inhibit phosphorylation of the NUAK1 substrate MYPT1, induced a ~50% reduction in the population of cells in S-phase (Figure 6A).

We also studied the impact that inhibiting NUAK1 had on the mitotic population of asynchronous U2OS cells over a 1000 min time course. This revealed that both WZ4003 and HTH-01-015 inhibitors markedly restricted cells from entering into mitosis to a similar extent as treatment as the RO3306 CDK1 inhibitor. After 600 min, no mitotic cells were detected following WZ4003 and HTH-01-015 treatment (Figure 6B). This mitotic progression defect could be a result of a defect in the highly regulated S-phase or the DNA replication phase of the cell cycle where NUAK1 might be playing a vital role.

We also observed that U2OS cells overexpressing the NUAK1[S476A+S480A] mutant proliferated at nearly 2-fold-higher rates than U2OS cells expressing WT NUAK1 or control cells not overexpressing NUAK1 (Figure 6C). We also found that overexpression of the NUAK1[S476A+S480A] mutant in U2OS cells induced a significant ~30–50% increase in population of mitotic cells compared with control U2OS cells that overexpress WT NUAK1 (Figure 6D).

### Evidence that NUAK1 regulates PLK1 T-loop phosphorylation

**Figure 7:**
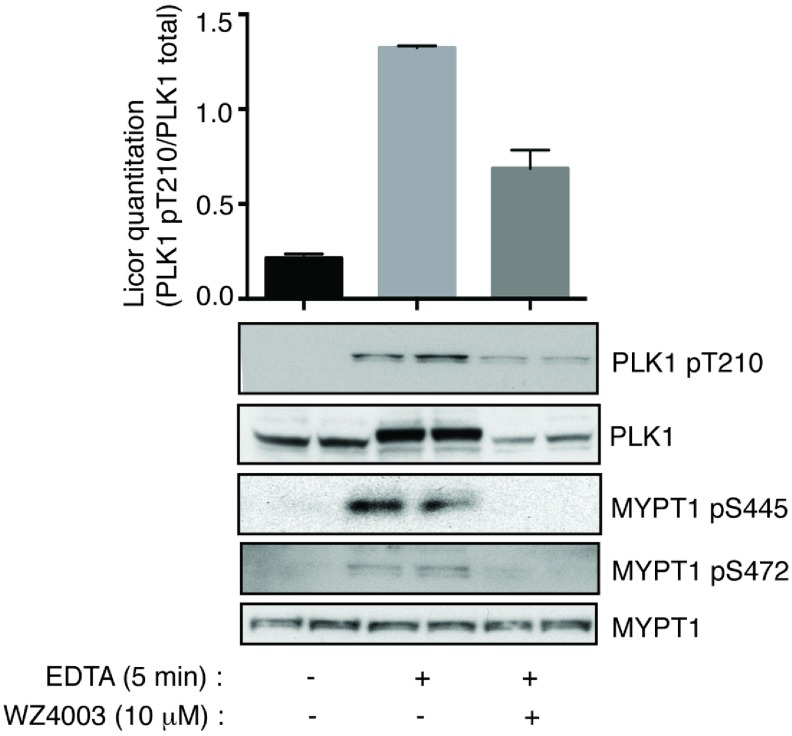
NUAK1 regulated PLK1 T-loop Thr^210^ phosphorylation HEK-293 cells were treated in the absence (DMSO) or presence of 10 μM of WZ4003 over 8 h. Cell medium was then replaced with either normal DMEM (−) or EDTA/PBS-based cell dissociation buffer (+) containing the same concentration of WZ4003 or DMSO that the cells were previously incubated in. Cell detachment was induced with gentle tapping of the plates followed by gentle centrifugation at 70 ***g*** for 3 min. Cells were rapidly lysed either by scraping for (−) cells or by resuspending in lysis buffer for (+) cells and the lysates were subjected to immunoblotting with the indicated antibodies. Results shown in the histogram are means±S.D.

As discussed in the Introduction section, PP1β^MYPT1^ binds to and inhibits PLK1 by dephosphorylating the T-loop residue (Thr^210^) [14]. The ability PP1β^MYPT1^ to bind to and dephosphorylate and hence inactivate PLK1 is dependent upon phosphorylation of MYPT1 at Ser^473^ by the CDK1, which creates a binding site for the PLK1 Polo-box domains [14]. As Ser^473^ lies adjacent to the NUAK1 phosphorylation site (Ser^472^) that controls 14-3-3 binding [6], we decided to explore whether NUAK1 could indeed influence PLK1 T-loop phosphorylation. We treated HEK-293 cells with EDTA to induce cell dissociation, a condition that has previously been shown to promote phosphorylation of MYPT1 by NUAK1 [6]. Immunoblotting with phospho-specific antibodies confirmed that EDTA treatment induced phosphorylation of MYPT1 at Ser^445^ and Ser^472^ (Figure 7). Strikingly, we observed that EDTA also induced a significant stimulation of PLK1 phosphorylation at Thr^210^ that was accompanied by marked electrophoretic band shift in PLK1 protein. Treatment of cells with the WZ4003 dual NUAK1/NUAK2 inhibitor prior to stimulation with EDTA, inhibited phosphorylation of Thr^210^ as well as the electrophoretic mobility shift of PLK1 protein (Figure 7).

## DISCUSSION

Our data demonstrate that NUAK isoforms are novel substrates for PLK1 and SCF^βTrCP^ E3 ubiquitin ligase complex. Our findings suggest that PLK by phosphorylating NUAK1 at Ser^476^ and Ser^480^ triggers interaction with the SCF^βTrCP^ complex resulting in ubiquitylation and degradation of NUAK1. This model is supported by NUAK1 expression being increased following inhibition of PLK (with BI2536 or GSK461364), mutation of Ser^476^/Ser^480^ or inhibition of the SCF complex (with MLN4924). We also demonstrate that in phases of the cell cycle where PLK1 is most active (G_2_–M), NUAK1 levels are low and *vice versa* when PLK1 is less active (late G_1_ to S), NUAK1 expression increases. Ser^476^ and Ser^480^ are highly conserved and located within the C-terminal domain of NUAK isoforms. To our knowledge Ser^476^/Ser^480^ have not been previously been documented (http://www.phosphosite.org/).

Phosphorylation of a number of other substrates by PLKs also triggers SCF^βTrCP^ binding thereby leading to ubiquitylation and degradation of the target. These include the Bora-activating subunit of the aurora A kinase [31,32], the Wee1 kinase regulator of mitotic entry [23], the mitotic regulator Emi1 (early mitotic inhibitor 1) [44,45], the DNA replication checkpoint regulator claspin 1 [46,47], the Fanconi's anaemia group ATP-dependent RNA helicase FANCM (Fanconi's anaemia complementation group M) [48] and HSF1 (heat-shock transcription factor 1) [49]. The phosphodegron motif that PLK1 phosphorylates in these proteins is similar to the ESGYYS motif encompassing Ser^476^ and Ser^480^ in NUAK1 as well as the equivalent residues in NUAK2 isoforms (Figure 2E). This phosphodegron motif is not conserved in any other LKB1-activated AMPK or AMPK-related kinases, indicating that the polo kinase-mediated phosphorylation and subsequent SCF^βTrCP^ ubiquitylation is unique to NUAK isoforms. Consistent with this we found that βTrCP only interacts with NUAK1 and NUAK2, but not with the other eight LKB1-activated AMPK family members investigated (Figure 1C).

For most PLK substrates, prior phosphorylation by a CDK isoform within a serine/threonine–proline motif creates a docking site that is recognized by a pair of C-terminal polo-box domains [33,34,50]. Our finding that treatment of cells with roscovitine suppresses binding of PLK1 to NUAK1 leading to increased expression (Figures 4A and 4C), supports the idea that CDK phosphorylation primes NUAK1 for PLK phosphorylation. Inspection of the NUAK1 phosphorylation sites (Supplementary Figure S2), led to the identification of Ser^445^ as the potential CDK phosphorylation site and PLK-binding residue. This is likely to comprise the CDK phosphorylation site as its mutation to alanine inhibits PLK1 and hence βTrCP binding to NUAK1 as well as calyculin A-mediated degradation (Figures 4E–4G).

**Figure 8:**
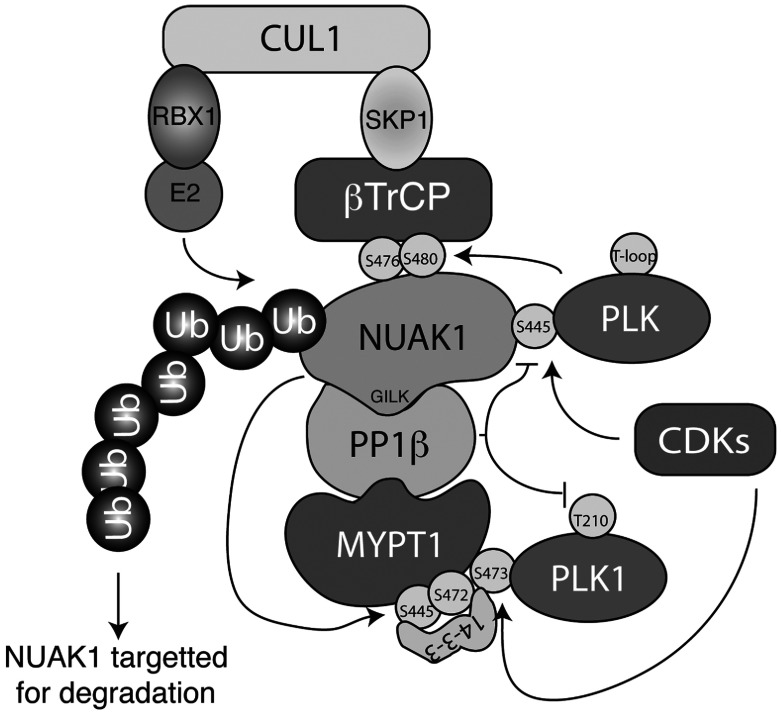
Schematic representation of how NUAK1 expression is regulated by PLK and SCF^βTrCP^ NUAK1 interacts with and phosphorylates PP1β^MYPT1^ myosin phosphatase complex at Ser^445^ and Ser^472^, which promotes 14-3-3 binding. In the S/G_2_-phase of the cell cycle NUAK1 and MYPT1 are primed by CDKs, which creates a docking site for PLK. PLK1 interacts with MYPT1 at Ser^473^ which leads to inactivation of PLK1 upon PP1β^MYPT1^-mediated dephosphorylation of Thr^210^ at the PLK1 activation loop. PLK docking on to NUAK1 at Ser^445^ leads to phosphorylation of NUAK1 at Ser^476^ and Ser^480^, which promotes interaction of NUAK1 with SCF^βTrCP^ E3 ubiquitin ligase complex that triggers ubiquitylation and degradation of NUAK1 in the G_2_/M-phase of the cell cycle. PP1β^MYPT1^ negatively regulates PLK–NUAK1 interaction by possibly dephosphorylating the CDK priming site on NUAK1 and hence protecting NUAK1 from degradation. RBX1, ring-box 1, E3 ubiquitin protein ligase.

NUAK isoforms possess three highly conserved GILK motifs that interact directly with the PP1β subunit of the PP1β^MYPT1^ complex [6]. Our findings suggest that inhibiting binding of NUAK1 to PP1β^MYPT1^ by ablating the GILK motifs significantly enhances βTrCP binding to NUAK1 (Figure 2H). This suggests that PP1β^MYPT1^ acts to dephosphorylate either the PLK1-binding CDK-primed site (Ser^445^) on NUAK1 and/or the βTrCP-binding residues (Ser^476^ to Ser^480^) (Figure 8). PLK1 has also been reported to interact with PP1β^MYPT1^, which leads to dephosphorylation of the PLK1 T-loop (Thr^210^) residue and thereby PLK1 inactivation [14,51]. Binding of PLK1 to MYPT1 is triggered following phosphorylation of the MYPT1 subunit by CDK1 at Ser^473^ [14]. Interestingly, one of the key NUAK1 phosphorylation sites on MYPT1, namely Ser^472^, lies adjacent to Ser^473^ that controls PLK1 binding to MYPT1. Phosphorylation of Ser^472^ triggers 14-3-3 isoforms binding to MYPT1 [6]. Our data indicate that subjecting HEK-293 cells to a condition that promotes endogenous NUAK1 to phosphorylate MYPT1, namely EDTA-induced cell detachment, leads to increased Thr^210^ phosphorylation of PLK1. This is also accompanied by an electrophoretic band shift, suggesting that stoichiometry of phosphorylation is significant (Figure 7). This phosphorylation of Thr^210^ as well as the electrophoretic mobility shift is inhibited by treatment with the NUAK1/NUAK2 WZ4003 selective inhibitor. These data support the notion that NUAK1 may indeed play a critical role in modulating PLK1 activity through its ability to phosphorylate MYPT1. In future work it would be interesting to explore whether phosphorylation of MYPT1 by NUAK1 influences association of PLK1 with PP1β^MYPT1^ and whether 14-3-3 binding is involved. It would also be important to analyse whether the effects that NUAK1 inhibitors have on suppressing mitosis (Figure 6) and cell proliferation [15] are mediated through their ability to induce dephosphorylation and hence inactivation of PLK1.

Our data suggest that there is a remarkable interplay between NUAK1, PP1β^MYPT1^, SCF^βTrCP^, PLK and CDK components (Figure 8). Conditions that enhance the ability of NUAK to phosphorylate MYPT1 will increase PLK1 activity, by inhibiting its dephosphorylation by PP1β^MYPT1^ complex. However, once PLK1 activity is increased, this should result in phosphorylation of Ser^476^ and Ser^480^ of NUAK1, at least once CDKs are activated. This would in turn promote SCF^βTrCP^ binding and hence result in ubiquitylation and degradation of NUAK1. Reduced NUAK1 level and activity will result in increased activity of PP1β^MYPT1^ complex,_,_ which will then inhibit PLK1 by inducing dephosphorylation of the T-loop of PLK1.

A recent study reports that the LKB1 pathway plays a role in regulating the centrosome via NUAK1, PP1β^MYPT1^ and PLK1 [52]. Consistent with our hypothesis that inhibiting NUAK1 will lead to increased association of PP1β^MYPT1^ and PLK1, the authors of that study observed that shRNA-mediated knockdown of LKB1, that would be expected to inhibit NUAK1 activity, promoted the association of MYPT1 and PLK1 [52]. However, despite these data, the authors then go on to argue that NUAK1 promotes interaction of PLK1 and MYPT1 thereby inducing dephosphorylation of PLK1 [52]. This conclusion is the opposite of what we have observed (Figure 7), and is also not consistent with the observation that shRNA knockdown of LKB1 promotes association of MYPT1 with PLK1.

We also found that treatment of cells with structurally diverse highly selective NUAK1 inhibitors (WZ4003 and HTH-01-015 [15]) reduced by ~50% the population of cells in S-phase and also inhibited cells entering mitosis to the same extent as treatment with the RO3306 CDK1 inhibitor (Figures 6A and 6B). Overexpression of a NUAK1 mutant in which Ser^476^ and Ser^480^ are ablated also significantly accelerated cell proliferation (Figure 6C). The finding that NUAK1 is controlled by critical cell cycle regulators namely CDKs, PLK1 as well as SCF^βTrCP^, that orchestrates ubiquitylation and degradation of key cell cycle co-ordinators [19], further highlights the role that NUAK1 is likely to play a major role in controlling cell cycle. PP1β^MYPT1^ has also been implicated in mediating important roles in the cell cycle, by dephosphorylating PLK1 as well as other proteins predicted to regulate kinetochore, centrosomes and central spindle [14,51]. Although NUAK1 is degraded in the G_2_–M-phase of the cell cycle, we believe that NUAK1 is playing a vital role in the S-phase and hence inhibition of NUAK1 leads to defects in S-phase which progressively leads to mitotic defects as well. In future work it would fascinating to explore in more depth the interplay between NUAKs, PLK1, PP1β^MYPT1^ and SCF^βTrCP^ to better define the role that this system plays in regulating cell proliferation. Both BI2536 and GSK461364 potently inhibit PLK2 and PLK3 as well [30], so although we have primarily focused on PLK1 in the present study, in future work it would be interesting to explore roles of the other Polo kinase family members in regulating the phosphorylation of NUAK1. It would also be interesting to explore whether inhibiting or activating NUAK1/NUAK2 impacted on the T-loop phosphorylation and hence activity of these other PLK isoforms. It would also be important to identify the key substrates that NUAK1 phosphorylates other than MYPT1 to regulate the cell cycle.

## Online data

Supplementary data
