# Supplementary material for: Interplay between Polo kinase, LKB1-activated NUAK1 kinase, PP1βMYPT1 phosphatase complex and the SCFβTrCP E3 ubiquitin ligase
Source: Biochem J. 2014 Jun 26;461(Pt 2):233–45. doi: 10.1042/BJ20140408 (PMC4109838; doi:10.1042/BJ20140408)
Supplement: Supplementary data [file bj4610233add.pdf]

BJ Signal

Biochem. J. (2014) **461**, 233–245 (Printed in Great Britain) doi:10.1042/BJ20140408

online data

SUPPLEMENTARY ONLINE DATA

Interplay between Polo kinase, LKB1-activated NUAK1 kinase, PP1β^{MYPT1} phosphatase complex and the SCF$^{\beta TrCP}$ E3 ubiquitin ligase

Sourav BANERJEE*[1], Anna ZAGÓRSKA†, Maria DEAK*, David G. CAMPBELL*, Alan R. PRESCOTT‡ and Dario R. ALESSI*[1]

*MRC Protein Phosphorylation and Ubiquitylation Unit, College of Life Sciences, University of Dundee, Dow Street, Dundee DD1 5EH, U.K.
†Molecular Neurobiology Laboratory, Salk Institute for Biological Studies, La Jolla, CA 92037, U.S.A.
‡Division of Cell Signalling and Immunology, College of Life Sciences, University of Dundee, Dow Street, Dundee DD1 5EH, U.K.

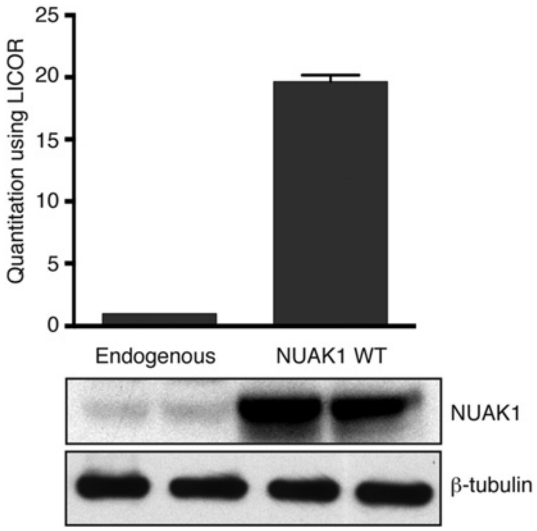

Figure S1 LI-COR quantitation of the overexpression of WT compared with endogenous NUAK1 expression

The Western blot signals for endogenous and overexpressed NUAK1 in U2OS Flp/In stable cells were quantified using LI-COR Odyssey technology. β-Tubulin was used as a loading control. The background signal was subtracted and then the band intensity of overexpressed NUAK1 was divided by the signal for the endogenous NUAK1 protein (in duplicates). Data are means±S.D. relative to the expression levels of the endogenous protein.

Biochemical Journal www.biochemj.org

[1] Correspondence may be addressed to either of these authors (s.y.banerjee@dundee.ac.uk or d.r.alessi@dundee.ac.uk).

© 2014 The Author(s)
This is an Open Access article distributed under the terms of the Creative Commons Attribution Licence (CC-BY) (http://creativecommons.org/licenses/by/3.0/) which permits unrestricted use, distribution and reproduction in any medium, provided the original work is properly cited.

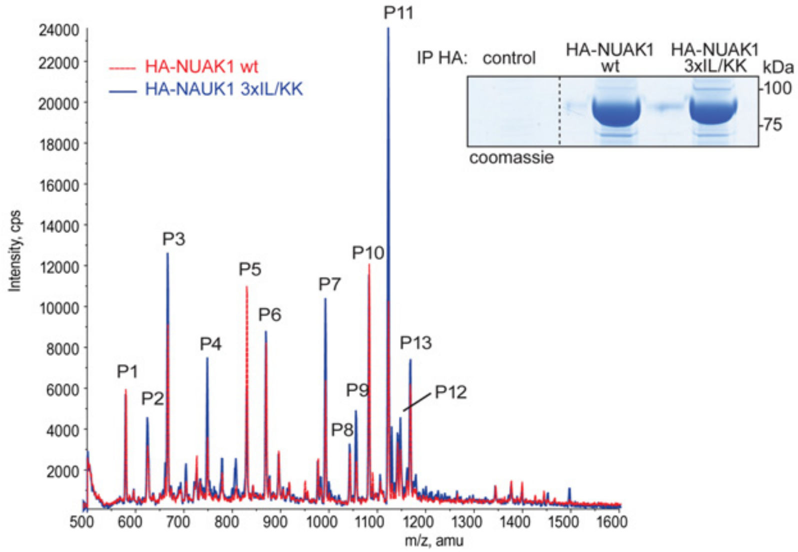

Peak	m/z	Peptide	Possible Phosphosite(s)
P1	580.9	R.YELQETLGK.G +1P	T60
P2	627.8	R.ESGYYSSPER.S +1P	**S480** or S481
P3	667.8	R.ESGYYSSPER.S +2P	**S476 or S480** or S481
P4	749.5	R.TGVLLPSSPEAEVPGKLSPK.Q +3P	S444 & S445 & S455
P5	831.1	R.TGVLLPSSPEAEVPGK.L +1P	S444 or S445
P6	871.0	R.TGVLLPSSPEAEVPGK.L +2P	S444 & S445
P7	993.5	K.ENDFAQSGQDAVPESPSK.L +1P	S388 or S390
P8	1043.7	R.TGVLLPSSPEAEVPGKLSPK.Q +1P	S455
P9	1056.2	R.SHSTGFIEGVVGPALPSTFK.M +1P	S411 or S413 or T414
P10	1083.7	R.TGVLLPSSPEAEVPGKLSPK.Q +2P	(S444 or S445) & S455
P11	1123.8	R.TGVLLPSSPEAEVPGKLSPK.Q +3P	T438 or S444 or S445 or S455
P12	1142.8	.MYPYDVPDYAEGAAAPVAGDRPDLGLGAPGSPR.E +1P	S22
P13	1168.8	K.FLQTFCGSPLYASPEIVNGR.P +1P	T211(T-loop)

*MYPYDVPDYA - HA-tag

Figure S2 Phosphorylation sites on NUAK1 WT and the 3IL/KK mutant

HEK-293 cells were transfected with HA–NUAK1 WT or a mutant with all three GILK motifs mutated to GKKK (3IL/KK). At 36 h after transfection cells were lysed and HA–NUAK1 was immunoprecipitated from 20 mg of cell lysates. Immunoprecipitates (IP) were separated on a polyacrylamide gel that was stained with colloidal Coomassie Blue. NUAK1 bands were then in-gel digested with trypsin and peptides were identified by LC-MS/MS on an ABSciex QTrap 4000 using precursor ion scanning for − 79 Da. Major peaks corresponding to identified phosphopeptides of NUAK1 WT (red) and NUAK1 3IL/KK (blue) are labelled P1–P13. Possible phosphorylation sites in the identified peptides are underlined.

© 2014 The Author(s)
This is an Open Access article distributed under the terms of the Creative Commons Attribution Licence (CC-BY) (http://creativecommons.org/licenses/by/3.0/) which permits unrestricted use, distribution and reproduction in any medium, provided the original work is properly cited.

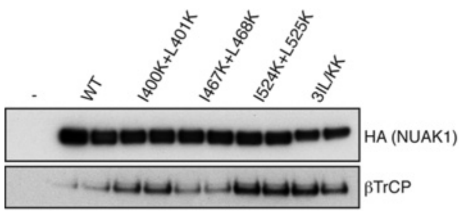

Figure S3 Effects of single IL/KK mutants on NUAK1–βTrCP interactions

HEK-293 cells were transfected with expression plasmids for the HA-tagged NUAK1 WT or indicated mutants. At 36 h post-transfection cells were lysed and HA-tagged proteins were immunoprecipitated from 1 mg of cell lysates. Immunoprecipitates (IP) were analysed by immunoblotting with the indicated antibodies.

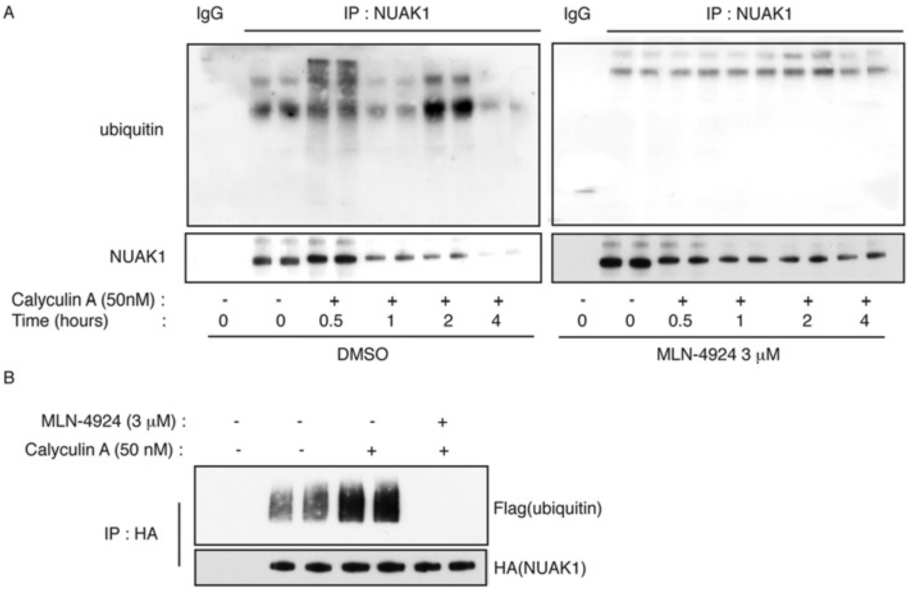

Figure S4 Calyculin A promotes polyubiquitylation and degradation of endogenous and overexpressed NUAK1, which can be reversed by MLN-4924

(**A**) Endogenous NUAK1 was immunoprecipitated from 1 mg of U2OS cells (lysed in the presence of NEM) treated with calyculin A (50 nM) and with or without pre-treatment of MLN-4924 (3 μM) over the indicated periods of time prior to lysis. MLN-4924 treatment was carried out for 30 min prior to calyculin A treatment. Immunoblotting was carried out to detect ubiquitin and NUAK1 levels in the immunoprecipitates. Pre-immune IgG was used as a control. (**B**) U2OS Flp/In NUAK1 WT cells were transfected with expression plasmids for FLAG-tagged ubiquitin. At 36 h post-transfection cells were treated with or without calyculin A (50 nM) for 15 min and with or without 30 min pre-treatment with MLN-4924 (3 μM) prior to lysis with lysis buffer containing 20 mM NEM. HA–NUAK1 was immunoprecipitated from 1 mg of cell lysates. Immunoprecipitates (IP) were analysed by immunoblotting with indicated antibodies.

© 2014 The Author(s)
This is an Open Access article distributed under the terms of the Creative Commons Attribution Licence (CC-BY) (http://creativecommons.org/licenses/by/3.0/) which permits unrestricted use, distribution and reproduction in any medium, provided the original work is properly cited.

Table S1 NUAK1-interacting proteins identified by mass spectrometry

The gel pieces shown in Figure 1(A) of the main text were digested with trypsin and proteins were identified by Orbitrap mass spectrometry. The identified proteins of importance that were present only in the NUAK1 immunoprecipitate (bands 7–12), but not in the control (bands 1–6), are listed. Mascot protein scores are shown.

Gel band	Protein	Symbol	Mass (Da)	Mascot score	Peptides
7	Probable ubiquitin C-terminal hydrolase FAF-X	USP9X	292720	3732	230
	Ubiquitin	UBB	18295	74	5
8	Myosin phosphatase regulatory subunit 1 (MYPT1)	PPP1R12A	115610	1134	92
	Myosin phosphatase regulatory subunit 2 (MYPT2)	PPP1R12B	110793	348	20
	Myosin-binding subunit 85 (MBS85)	PPP1R12C	85286	207	19
9	NUAK family Snf1-like kinase 1 (BAIT)	NUAK1	74772	5947	256
10	F-box/WD repeat containing protein 11	βTrCP2	61772	353	32
	F-box/WD repeat containing protein 1A	βTrCP1	66262	208	20
11	Serine/threonine protein phosphatase PP1β catalytic subunit	PPP1CB	37961	970	35
12	14-3-3 protein epsilon	YWHAE	29326	324	13
	S-phase kinase associated protein 1	SKP1	18817	46	2
	Ubiquitin	UBB	18295	56	2

Received 27 March 2014/1 May 2014; accepted 2 May 2014
Published as BJ Immediate Publication 2 May 2014, doi:10.1042/BJ20140408

© 2014 The Author(s)
This is an Open Access article distributed under the terms of the Creative Commons Attribution Licence (CC-BY) (http://creativecommons.org/licenses/by/3.0/) which permits unrestricted use, distribution and reproduction in any medium, provided the original work is properly cited.
